# Erythema Migrans-like COVID Vaccine Arm: A Literature Review

**DOI:** 10.3390/jcm11030797

**Published:** 2022-02-01

**Authors:** Gaia Fasano, Luigi Bennardo, Silvana Ruffolo, Maria Passante, Azzurra Gaia Ambrosio, Maddalena Napolitano, Eugenio Provenzano, Steven Paul Nisticò, Cataldo Patruno

**Affiliations:** 1 Department of Health Sciences, Magna Graecia University, 88100 Catanzaro, Italy; fasano.gaia@gmail.com (G.F.); mariapassante989@gmail.com (M.P.); azzurra.ambrosio@gmail.com (A.G.A.); steven.nistico@gmail.com (S.P.N.); cataldo.patruno@unicz.it (C.P.); 2 Mariano Santo Hospital, 87100 Cosenza, Italy; sruffolo@aocs.it (S.R.); eprovenzano0@gmail.com (E.P.); 3 Department of Health Sciences Vincenzo Tiberio, University of Molise, 86100 Campobasso, Italy; maddy.napolitano@gmail.com

**Keywords:** COVID Vaccine Arm (CVA), Spikevax^®^ Moderna (mRNA-1273) vaccine, Cominarty^®^ Pfizer/BioNTech’s (BNT162b2) vaccine, COVID-19, Erythema migrans-like

## Abstract

COVID Vaccine Arm (CVA) is an adverse drug reaction from mRNA vaccine for SARS-CoV-2. CVA is characterized by erythema and edema on the vaccination site (usually deltoid area) that appears from 5 to 10 days after vaccination and is sometimes associated with itching or pain. The exact etiology of CVA is still unclear, but delayed hypersensitivity against an excipient seems to play an essential role in the pathogenesis of the disease. This work performs a systematic literature review on CVA using three different databases containing articles published until 10 November 2021. The literature review includes eight papers reporting single cases or case series of CVA. Moreover, it also addresses, other cutaneous reactions following COVID 19 vaccinations as well as possible differential diagnosis. CVA migrans-like erythema is characterized by a ring-shaped rash in the injection area, which appears some days after the injection and disappears in about 10 days. This reaction may appear more rapidly in subsequent doses.

## 1. Introduction

The Coronavirus disease-2019 (COVID-19) was officially declared pandemic by the WHO on 11 March 2020, and the rapid vaccine development became a global priority [1].

The Food and Drug Administration (FDA) in December 2020 authorized the use of Cominarty^®^ Pfizer/BioNTech’s (BNT162b2) and Spikevax^®^ Moderna (mRNA-1273) COVID-19 vaccines in order to overcome the worldwide emergency [2].

COVID Vaccine Arm (CVA) is a recently observed transient skin reaction resulting from mRNA vaccination that affects approximately 2% of subjects who received the vaccine [3]. The emerging medical literature studying Moderna and Pfizer/BioNTech’s vaccines described CVA [4].

CVA is characterized by erythema and edema at the vaccination site and appears from 5 to 10 days after vaccination.

CVA can also appear in different body parts, even if not close to the injection site [5]. Moreover, CVA is commonly associated with pain or burning sensation, but it can be asymptomatic [5,6,7].

Rarely, the CVA eruption has been mistakenly diagnosed as cellulitis and a systemic antibiotic therapy was recommended as prophylaxis, in addition to the topical corticosteroid therapy [3].

**Figure 1:**
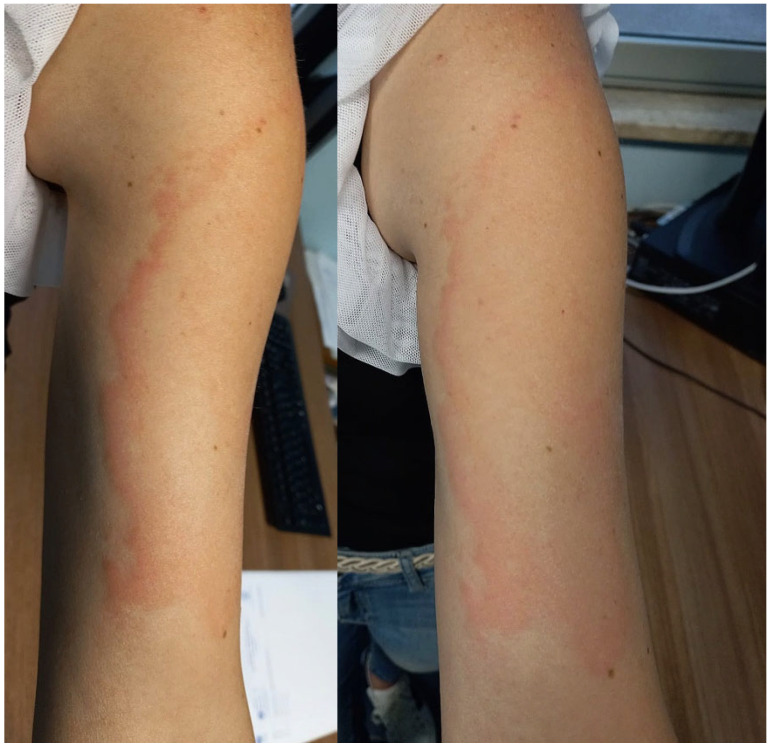
Erythematous, edematous, marginated Erythema migrans-like COVID Vaccine Arm eruption 7 days after the first dose of the Moderna vaccine.

Usually, CVA resolves spontaneously within a few weeks. However, some patients are treated with systemic antihistamines, and topical or oral glucocorticoids in order to relieve subjective symptoms [8] (Figure 1).

The etiopathogenesis of CVA is still unclear and a delayed hypersensitivity reaction induced by some vaccine components is currently considered the most likely hypothesis [9].

The reaction after the first administration is not a contraindication to the second administration. However, patients and health care professionals should be aware that this type of reaction may develop more rapidly after the second vaccine dose [10,11].

Given the high incidence of this reaction (up to 2% of vaccinated patients) and the relatively low number of reports, we performed a literature review about CVA, its differential diagnosis, and other cutaneous reactions caused by the COVID vaccine. The aim of the paper consists in improving the awareness of this reaction among clinicians and researchers.

## 2. Materials and Methods

The authors carried on a systematic literature review on CVA using the guidelines and the criteria established from the Preferred Reporting Items for Systematic Reviews and Meta-Analyses (PRISMA) 

Two independent researchers (L.B. and G.F.) performed a comprehensive literature search to identify relevant studies from 20 July 2021 up to 10 November 2021, with no temporal restriction, using the following databases: MEDLINE/PubMed (National Center for Biotechnology Information, NCBI), EMBASE (Ovid), and Google Scholar. The search string contained Medical Subject Headings (MeSH) and free-text terms. 

The research algorithm comprehended the following keywords: “COVID-19 vaccine arm”, “skin COVID-19 vaccine”, and “adverse skin reaction COVID-19 vaccine”. We screened all articles’ titles and abstracts containing such keywords. 

In addition, we also searched for citations included in the reference list of the selected articles. After eliminating the duplicates, the eligible articles were screened based on the title and the abstract. Finally, we analyzed the full text of the articles potentially suitable for inclusion in the systematic reviews. In case of discrepancies among authors, a third senior researcher (C.P.) decided whether to include or not one article.

## 3. Results

**Figure 2:**
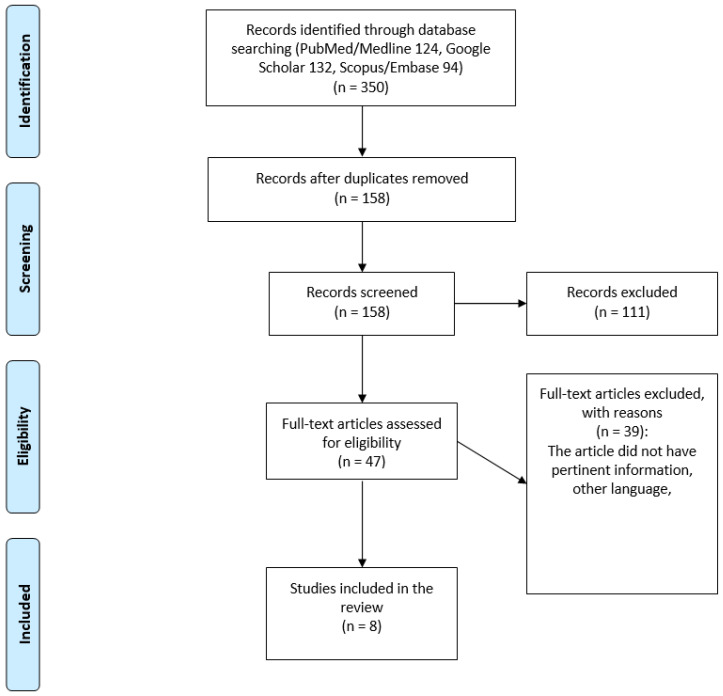
Articles selection flowchart.

The literature search identified 158 articles, 111 of which have been removed afterthe activity of screening of titles and abstracts. The full text of the remaining 47 papers was assessed for inclusion, and 2 articles were excluded as in non-english language; finally, 8 papers met the inclusion criteria; and, thus, they were included in the review. The article selection flow chart (Figure 2) summarizes the search strategy adopted in this study. We analyzed 5 case series and 3 case reports for a total of 29 patients.

All the patients reported a reaction near the injection site after the complete resolution of the other local and systemic symptoms associated with vaccination.

The reaction was reported both after Moderna vaccine (89.7%) and Pfizer/BioNTech’s vaccine (10.3%) after first (89.7%) or second dose (10.3%) with a median onset on day 7.7 (range 3 to 11).

The median age (year) of patients was 56 (range 31 to 86), while 89.7% of patients were female.

In medical history, 9 patients (31%) reported allergic predispositions such as pharmacological allergy, rhinitis, urticaria, angioedema, contrast allergy, wasp allergy, atopic history, and others. Moreover, patiens reported other sporadic comorbidities, probably not correlated to that reaction, such as psoriasis, atrial fibrillation, hypercholesterolemia, hypothyroidism, breast and ovarian cancer, melanoma, non-melanoma skin cancer, chronic obstructive pulmonary disease (COPD), and pulmonary hypertension.

Almost all patients reported erythema, red plaque, pruritus, warmth, swelling, scaling, and pain.

Only 24.1% of patients reported systemic symptoms such as fever, headache, myalgia, chills, or other cutaneous symptoms not close to the injection area such as papules on the palm and fingers or urticarial plaques on the elbows. One patient reported lymphadenopathy, while another patient reported tachycardia and hypertension.

The rash diameter was detected only for 16 patients with a median of 9.9 cm (range 4 to 19.5).

Skin biopsy was performed only in 4 patients and reported a focal spongiosis with vacuolar alteration and few lymphocytes in the epidermis; an inflammatory perivascular infiltrate was found in the dermis with lymphocytes and some histocytes, eosinophilic granulocytes, and neutrophils.

Almost all patients did not undergo any therapy; some patients (41.4%) used topical corticosteroids such as clobetasol propionate 0.05%, mometasone furoate 0.01%, methylprednisolone-aceponate 0.1%, hydrocortisone 1%, triamcinolone 0.1% or other topical agents such as diclofenac, diphenhydramine hydrochloride 1%.

Only 7 patients (24.1%) used oral antihistamines such as cetirizine 10 mg, loratadine 10 mg, desloratadine, diphenhydramine 25 mg, diphenhydramine 25 mg, famotidine 20 mg.

The rash and the other cutaneous symptoms resolved spontaneously or thanks to the treatment about 4 days on average after the onset (range 1 to 7). Table 1 and Table 2 report all the studies selected for this review as well as patients’ characteristics.

## 4. Discussion

The first 4 cases of CVA after the first dose of Moderna vaccine have been described by Wei et al. [8].

Anshari et al. [9] evidenced the case of a 56-old woman who manifested CVA 3 days after booster vaccination using Moderna vaccine. The symptoms disappeared with surface cooling and compression stocking 8 days later. Histological examination showed spongiosis with vacuolar alterations and perivascular inflammatory infiltrate in periadnexal areas, on superficial and deep dermal plexi, and subcutaneous fat, consisting of lymphocytes and some histiocytes with few intravascular neutrophils. Eosinophils were not present as signs of vascular wall damage. The biopsy result was consistent with delayed-type hypersensitivity.

Zengarini et al. [12] reported the case of a 63-years-old female who presented flat and targetoid erythema without other local symptoms. This manifestation was suspected to be an erythema migrans due to the marginate clinical aspect and to the fact that the patient was from an endemic area for Borrelia burgdorferi. The patient was vaccinated using a first dose of the Moderna vaccine 5 days prior to the appearance of the rash. The rash did not appear on the vaccine injection site, but in a different body area. In literature, Blumenthal et al. described delayed large local reactions even far from the injection site [5]. These reactions appeared on the same arm where the vaccine was injected, but away from the puncture site [5]. Wei et al. described it as “COVID vaccine arm” because it showed up some days after the first dose of vaccine [8]. COVID vaccine arm is similar to the insect bites’ reactions. However, it can be distinguised through clinical history [15].

Kempf et al. [13] analyzed skin biopsy of 3 patients who had erythema on the left arm 6–7 days after the first dose of Moderna vaccine. Histology revealed epidermal changes with spongiosis and exocytosis of a few lymphocytes. Small lymphocytes (CD4+ and CD8+) and eosinophilic granulocytes have been reported in the dermal perivascular inflammatory infiltrate. The immunophenotypic profile revealed the presence of CD3+ T cells, CD4+ T cells, Tregs, plasma cells, and PDCs. Eosinophils were present in a variable number. The Authors concluded that the COVID vaccine arm has an immunological pattern which can be interpreted as a delayed-type hypersensitivity reaction [13].

Barriere et al. [11] described the case of a 76-year-old female with ovarian neoplasia who developed oedema without erythema, pain, and a 2 cm painless axillary lymphadenopathy, 5 days after the second dose of Pfizer/BioNTech’s vaccine. PET/FDG imaging showed a complete metabolic response to the peritoneal target, but hypermetabolism in the lymph node and in the deltoid muscle was found [11]. Vaccination is the cause of a transient locoregional inflammatory reaction with inflammation of lymph nodes that can induce positive findings on FDG-PET [11]. Is important to know this local reaction to avoid invasive diagnostic and therapeutic procedures [11].

Gregoriou et al. described 4 cases of CVA, one of which was after Pfizer/BioNTech’s vaccine. [4].

In healthcare workers, D. Fernandez-Nieto et al. [3] analyzed the skin manifestation of the ^®^Pfizer/BioNTech vaccine. The researchers analyzed 4775 subjects who received the Pfizer/BioNTech vaccination; 18% of them experienced general side effects. 2% (103 people) had delayed skin reactions: 47.6% (49/193) after the first vaccine dose, 52.4% (54/103) after the second dose. 32.7% (16/49) had recurrance after the second dose. The reaction duration was variable: in 22.3% of patients, the reaction resolved in less than 8 h; in 26.2% of patients the reaction lasted between 8 and 24 h; in another 36.9% of workers it lasted between 48 and 72 h and only in 13.6% of cases it lasted more than 72 h. 68% of patients experienced itch (70 patients); 4.9% presented local or disseminated reactions (5 patients). None of the patients developed an anaphylactic reaction. A skin biopsy with histological examination was performed on a patients who presented an erythematous targetoid patch on the injection site. The biopsy showed a superficial and deep perivascular lymphocytic infiltrate with dilated vessels and intraluminal neutrophils. Immunohistochemistry for the SARS-CoV-2 spike 1A9 protein was negative.

Recently, a case series of delayed large local reactions to the Moderna vaccine [5], including 12 cases, showed that the average onset of reaction after the first dose was about day 8 and the resolution average time was 6 days. Six patients experienced similar reactions also after the second dose with an average onset of 2 days. The reaction onset after the second dose was earlier with respect to the first dose [5]. Another study showed that patients who experienced CVA-both after the first and second vaccine dose-experienced symptoms faster after the second than the first injection (1–3 days) [16].

### 4.1. Cutaneous Reactions after COVID-19 Vaccination

Both COVID-19 infection and COVID-19 vaccines can cause multiple cutaneous reactions [17].

In clinical trials of 11 authorized COVID-19 vaccines, the most common adverse cutaneous reactions were local injection site reactions: erythema, pain, itching, swelling, pruritus, and tension on the injection site; the symptoms resolved over the next 24 to 48 h [18]. In phase III of Moderna clinical trials, among 15,185 participants who got vaccination 228 (1.5%) developed, within 7 days after the first dose, delayed large local reactions such as erythema, induration, and tenderness [19]. After the second dose, 68 (0.2%) of participants developed delayed large local reactions [19]. Less common observed reactions are: allergic, atopic and contact dermatitis, eczema, exfoliative rash, and vesicular rash [19].

Urticaria, angio-oedema, and anaphylaxis are type I hypersensitivity reactions due to allergy to some ingredients; they are not very common although theycan be severe [17].

Another type of reaction induced by Moderna vaccine is the erythema multiforme [20,21,22]. Muhamad Khalid et al. [23] reported the case of a patient who developed a mild rash 2 weeks after the first dose that resolved without any treatment. After the second dose, the patient developed large blisters and redness to the anterior chest, genitalia, bilateral hands, and bilateral lower feet without facial or mucosal involvement. There were no other associated systemic symptoms. The biopsy showed the presence of eosinophils that were suggestive of drug-induced erythema multiforme. Likely, the cause of it is due to the temporal relationship between vaccination and rash development.

Ackerman M et al. [24] described a morbilliform rash (maculopapular, pruritic exanthem) that erupted over 30% of a patient’s body. The rash developed on the face, trunk, upper extremities, sparing oral and genital mucosa, followed by a systemic manifestation with liver injury. Rash and liver damage enzymes improved after corticosteroid treatment.

Type IV hypersensitivity reactions on previous radiation sites are also described [17].

Soyfer et al. [25] described a dermatitis in previously irradiated skin sites of 2 patients after Pfizer/BioNTech’s vaccination.

Delayed inflammatory reactions in the site of dermal hyaluronic acid fillers have been described; Munavalli et al. [26] described fifteen cases: 11 after Moderna vaccine and 4 after Pfizer/BioNTech’s vaccine. The areas treated with fillers showed up swelling and inflammation 24–48 h after the vaccination. These reactions have also been observed after other vaccines, such as influence and in patients with COVID-19 infection [17]. Ethiopathological mechanism is probably related to the expression of angiotensin-converting enzyme (ACE) receptors in adipose tissue where the fillers have been injected [26].

The vaccine-induced spike protein would determine the stimulation of ACE2 which is its target. This would induce stimulation of CD8 in particular and subsequent Th1 inflammatory response. This mechanism could be confirmed by similar reactions observed in the granuloma from vaccination against TB with BCG in some healthcare professionals, always after administration of the two mRNA vaccines [27]. ACE blockers is the therapy to prefer over the corticosteroid, as the latter can reduce vaccine efficacy [17].

Lopatynsky-Reyes et al. [27] described a local skin inflammation in scar sites due to previous BCG vaccination accompanied by headache, myalgia, malaise, and arthralgia one day following the second dose of both Pfizer/BioNTech’s and Moderna vaccination.

Chilblain-like lesions have been observed after both COVID-19 infections and the COVID-19 vaccine. These lesions appear as erythematous, violaceous papules, and macules on the hands and feet; treatment with topical steroids can reduce the symptoms [18]. Qisi et al. [18] described 10 cases of pernio and chilblains: 6 associated with Pfizer/BioNTech’s vaccine and 4 associated with Moderna vaccine.

Lichen planus, herpes simplex, reactivation of herpes zoster, pityriasis rosea (reactivation of HHV 6 and HHV 7), maculopapular rash, swelling of the face, erythromelalgia, and petechial rash were very rare. No severe adverse reactions were reported [18,28,29,30].

Leukocytoclastic vasculitis, lupus erythematosus, and immune thrombocytopenia are possible immuno-mediated skin reactions reported [17].

### 4.2. Differential Diagnosis

CVA must be differentiated from other skin eruptions.

CVA associated with systemic symptoms has been misdiagnosed for cellulitis [14]. It is possible to distinguish CVA from cellulitis based on the time of the onset (1 week vs. 5 days), absence of systemic symptoms, resolution time of approximately 4–5 days, spontaneous resolution, or rapid response to treatment with topical corticosteroids and antihistamines. Pruritus is also commonly found in CVA manifestations. [9,14].

Halperin, et al. [12] defined the diagnostic criteria to differentiate between cellulitis and local reaction post-vaccination. Cellulitis had three main symptoms: local pain, erythema, induration/swelling, and warmth [9]. Furthermore, the response to antibiotics can help confirm the diagnosis [9].

Montjoye et al. [31] described the case of eosinophilic cellulitis or Wells syndrome after Pfizer/BioNTech’s vaccine. A 71-year-old woman presented on the right arm a painful eruption the day after the second dose. After 12 days, this eruption became erythematous and swollen with vesciculobullous lesions and erosions without fever. The suspecting cellulitis, a combination of amoxicillin and clavulanic acid was administered. Blood tests showed hypereosinophilia and slightly elevated C-reactive protein levels. Histological examination showed spongiotic dermatitis with dermal infiltrate (lymphocytes, histiocytes and eosinophils) and epidermal vesicles.A diagnosis of eosinophilic cellulitis was made. Vaccination is probably a triggering factor for this hypersensitivity reaction [31].

Ashley et al. [32] reported pediatric eosinophilic cellulitis 10 days after receiving tetanus, diphtheria, pertussis, and polio and measles, mumps, rubella, and varicella vaccines. The patch test showed a 1+ reaction to aluminum, hydroxide and neomycin at 96 h, [32]. HoweverPfizer/BioNTech’s vaccine does not contain these components, and further studies are needed to investigate the underlying etiopathogenetic cause.

We can distinguish CVA from erythema migrans through the absence of: recent tick bites, systemic neurological symptoms, IgM and IgG anti-Borrelia, and the rapid response to treatment with topical corticosteroids and antihistamines [12].

## 5. Conclusions

Vaccines are powerful and essential weapons against COVID-19 emergency. Skin side effects are generally minor and self-limited and should not discourage vaccination.

CVA is a mild possible side effect and not a contraindication for the second dose [8]. However, patients and health care should be aware that this reaction may develop more rapidly after the second dose [10].

CVA migrans-like erythema is an adverse reaction characterized by a ring erythematous rash in the injection area, which occurs some days after the vaccine and resolves in about 10 days [5]. CVA should be distinguished from the more common local reactions which are observed one day after vaccination and which last 2 to 3 days [33]. It is essential to know the possible heterogeneity of CVA and educate the general practitioners to recognize, through a detailed analysis of family and personal history, all the various manifestations associated with the Moderna vaccine.

## Figures and Tables

**Table 1:** Selected studies.

Author	Study Type	Number of Patients	Type of Vaccine
Wei et al. [8]	Case series	4	Moderna mRNA-1273
Anshari et al. [9]	Case report	1	Moderna mRNA-1273
Zengarini et al. [12]	Case report	1	Moderna mRNA-1273
Kempf et al. [13]	Case series	3	Moderna mRNA-1273
Barriere et al. [11]	Case report	1	Cominarty (Pfizer/BioNTech)
Gregoriou et al. [4]	Case series	4	Moderna mRNA-1273 and Cominarty (Pfizer/BioNTech)
Blumenthal et al. [5]	Case series	12	Moderna mRNA-1273
Lindgren et al. [14]	Case series	3	Moderna mRNA-1273 and Cominarty (Pfizer/BioNTech)

**Table 2:** Characteristics of patients included in the review.

Study and Patient’s Number	Age	Gender	Medical History or Allergies	Type of Vaccine	Days after Vaccination	Localized Symptoms	Rash Diameter	Systemic Symptoms	Skin Biopsy	Therapy	Outcome
Wei et al. [1]	74	Female	No	Moderna mRNA-1273	8 days after first dose	Pruritus, erythematous plaque, mild scaling	15 cm	No	No	Topical clobetasol proprionate 0.05% cream and oral cetirizine 10 mg	Partial resolution after 1 week
Wei et al. [2]	62	Female	No	Moderna mRNA-1273	8 days after first dose	Pruritus, erythematous plaque, edema, warmth	Not reported	No	No	Mometasone furoate 0.01% ointment, diphenhydramine hydrochloride 1% cream, oral loratadine 10 mg	
Wei et al. [3]	54	Female	No	Moderna mRNA-1273	7 days after first dose	Erythematous rash	Not reported	No	No	No	Resolving spontaneously in 4 days
Wei et al. [4]	72	Female	Psoriasis, atrial fibrillation, hypercholesterolemia, hypothyroidism	Moderna mRNA-1273	10 days after first dose	Pruritus, Erythematous plaque, warmth	14 cm	No	No	No	Resolving spontaneously in 2 days
Anshari et al. [1]	56	Female	Breast cancer, atopic history with eczema and allergic rhinitis, thalassemia trait	Moderna mRNA-1273	3 days after second dose	Swell, redness, warm, pain	Not reported	No	Focal spongiosis and vacuolar alteration in the epidermidis. Inflammatory infiltrate perivascular in the dermis with lymphocytes and some histocytes and neutrophils.	Surface cooling and compression	Resolution after 5 days
Zengarini et al. [1]	63	Female	No	Moderna mRNA-1273	5 days after first dose	Flat and targetoid erythema with bull’s eye aspect	Not reported	No	No	Surface cooling and compression	Resolution after 2 days
Kempf et al. [1]	84	Male	Melanoma, non-melanoma skin cancer	Moderna mRNA-1273	7 days after first dose	Erythema	Not reported	No	Focal spongiosis and exocytosis of a few lymphocytes in the epidermidis. Inflammatory infiltrate perivascular in the dermis with lymphocytes and some eosinophilic granulocytes and neutrophils.		
Kempf et al. [2]	86	Female	Non-melanoma skin cancer	Moderna mRNA-1273	7 days after first dose	Erythema	Not reported	No	Focal spongiosis and exocytosis of a few lymphocytes in the epidermidis. Inflammatory infiltrate perivascular in the dermis with lymphocytes and some eosinophilic granulocytes and neutrophils.		
Kempf et al. [3]	81	Female	Non-melanoma skin cancer, eczema of hand	Moderna mRNA-1273	7 days after first dose	Erythema	Not reported	No	Focal spongiosis and exocytosis of a few lymphocytes in the epidermidis. Inflammatory infiltrate perivascular in the dermis with lymphocytes and some eosinophilic granulocytes and neutrophils.		
Barriere et al. [1]	76	Female	Ovarian Neoplasia	Comirnaty Pfizer-Biontech	5 days after the second dose	Inflammatory edema, pain	Not reported	No	No		
Gregoriou et al. [1]	733	Female	No	Moderna mRNA-1273	9 days after first dose	Erythematous papules with red plaque, scaling, pruritus	7 cm	No	No	Topical methylprednisolone-aceponate 0.1% cream and desloratadine	Resolution after 4 days
Gregoriou et al. [2]	74	Female	Chronic obstructive pulmonary disease (COPD), pulmonary hypertension	Cominarty (Pfizer/BioNTech)	8 days after second dose	Erythematous plaque	Not reported	No	No	Topical methylprednisolone-aceponate 0.1% cream	Resolution after 3 days
Gregoriou et al. [3]	51	Female	No	Moderna mRNA-1273	9 days after first dose	Erythematous plaque	Not reported	No	No	Topical methylprednisolone-aceponate 0.1% cream	Resolution after 3 days
Gregoriou et al. [4]	53	Female	No	Moderna mRNA-1273	11 days after first dose	Erythematous plaque	8 cm	No	No	Topical mometasone furoate 0.1% cream	Resolution after 5 days
Blumenthal et al. [1]	37	Female	No	Moderna mRNA-1273	8 days after first dose	Annular papules, pruritus	9 cm	No	No	No	
Blumenthal et al. [2]	61	Female	Contrast allergy	Moderna mRNA-1273	8 days after first dose	Edematous plaque, pruritus, warmth	10 cm	No	No	Topical clobetasol propionate 0.05% cream	
Blumenthal et al. [3]	45	Female	Rhinits, penicillin allergy	Moderna mRNA-1273	8 days after first dose	Edematous plaque, pruritus, pain	14 cm	Fatigue, headache, myalgias, chills	No	Topical hydrocortisone 1% cream, diphenhydramine 25 mg	
Blumenthal et al. [4]	31	Female	Urticaria, rhinits	Moderna mRNA-1273	8 days after first dose	Erythematous plaque, pruritus	5 cm	Lymphadenopathy	No	Topical triamcinolone 0.1% cream, diclofenac 1% topical gel, cetirizine 10 mg	
Blumenthal et al. [5]	40	Female	No	Moderna mRNA-1273	4 days after first dose	Erythematous plaque, pruritus, pain	13 cm	Papules on the palm and fingers. Headache, fatigue, fever	No	No	
Blumenthal et al. [6]	43	Male	No	Moderna mRNA-1273	9 days after first dose	Erythematous plaque, pruritus, pain, warmth	12.5 cm	Urticarial plaques on the elbows	No	Diphenhydramine 25 mg, famotidine 20 mg	
Blumenthal et al. [7]	38	Female	Wasp allergy	Moderna mRNA-1273	9 days after first dose	Erythematous plaque, pain	7 cm	No	No	Loratadine 10 mg	
Blumenthal et al. [8]	49	Female	Idiopathic urticaria	Moderna mRNA-1273	8 days after first dose	Indurated plaque, pruritus, pain, burning, warmth	4 cm	No	No	No	
Blumenthal et al. [9]	41	Female	No	Moderna mRNA-1273	10 days after first dose	Indurated plaque, pruritus, warmth	7.5 cm	Fatigue	No	No	
Blumenthal et al. [10]	47	Male	Almond allergy, rhinits	Moderna mRNA-1273	11 days after first dose	Erythematous plaque, pain	7 cm	Fatigue, myalgias	No	No	
Blumenthal et al. [11]	52	Female	Angioedema,	Moderna mRNA-1273	8 days after first dose	Erythematous plaque, swelling, pain	19.5 cm	Tachycardia, hypertension	No	No	
Blumenthal et al. [12]	46	Female	Penicillin allergy	Moderna mRNA-1273	9 days after first dose	Erythematous plaque, pruritus	7 cm	Headache	No	No	
Lindgren et al. [1]	60	Female	No	Moderna mRNA-1273	6 days after first dose	Erythematous papules with pruritus, swollen, pain	Not reported	No	No	Topical clobetasol 0.05% cream	Resolution after 1 day
Lindgren et al. [2]	44	Female	No	Cominarty (Pfizer/BioNTech)	7 days after first dose	Erythema, pain, pruritus, sweeling	Not reported	Fever, chills, headache, myalgias	No	Topical triamcinolone 0.1% cream	Resolution after 2 days
Lindgren et al. [3]	33	Female	No	Moderna mRNA-1273	7 days after first dose	Erythema, pain, pruritus, swelling	Not reported	No	No	Topical hydrocortisone 1% cream	Resolution after 4 days

## Data Availability

The data presented in this study are available on request from the corresponding author. The data are not publicly available due to privacy.
